# Whole-genome sequencing of a year-round fruiting jackfruit (*Artocarpus heterophyllus* Lam.) reveals high levels of single nucleotide variation

**DOI:** 10.3389/fpls.2022.1044420

**Published:** 2022-12-20

**Authors:** Tofazzal Islam, Nadia Afroz, ChuShin Koh, M. Nazmul Hoque, Md. Jillur Rahman, Dipali Rani Gupta, Nur Uddin Mahmud, Abdullah Al Nahid, Rashedul Islam, Pankaj K. Bhowmik, Andrew G. Sharpe

**Affiliations:** ^1^ Institute of Biotechnology and Genetic Engineering (IBGE), Bangabandhu Sheikh Mujibur Rahman Agricultural University (BSMRAU), Gazipur, Bangladesh; ^2^ Global Institute for Food Security (GIFS), University of Saskatchewan, Saskatoon, SK, Canada; ^3^ Department of Gynecology, Obstetrics and Reproductive Health, Bangabandhu Sheikh Mujibur Rahman Agricultural University (BSMRAU), Gazipur, Bangladesh; ^4^ Pomology Division, Horticultural Research Center, Bangladesh Agricultural Research Institute, Gazipur, Bangladesh; ^5^ Department of Biochemistry and Molecular Biology, Shahjalal University of Science and Technology, Sylhet, Bangladesh; ^6^ Bioinformatics Graduate Program, University of British Columbia, Vancouver, BC, Canada; ^7^ Cell Technologies and Trait Development, National Research Council of Canada, Saskatoon, SK, Canada

**Keywords:** BARI Kanthal-3, genome sequencing and assembly, heterozygosity, SCOS, SNPs, scaffolds, flowering gene orthologues

## Abstract

Jackfruit (*Artocarpus heterophyllus* Lam.) is the national fruit of Bangladesh and produces fruit in the summer season only. However, jackfruit is not commercially grown in Bangladesh because of an extremely high variation in fruit quality, short seasonal fruiting (June-August) and susceptibility to abiotic stresses. Conversely, a year-round high yielding (ca. 4-fold higher than the seasonal variety) jackfruit variety, BARI Kanthal-3 developed by the Bangladesh Agricultural Research Institute (BARI) derived from a wild accession found in Ramgarh of Chattogram Hiltracts of Bangladesh, provides fruits from September to June. This study aimed to generate a draft whole-genome sequence (WGS) of BARI Kanthal-3 to obtain molecular insights including genes associated with year-round fruiting trait of this important unique variety. The estimated genome size of BARI Kanthal-3 was 1.04-gigabase-pair (Gbp) with a heterozygosity rate of 1.62%. *De novo* assembly yielded a scaffolded 817.7 Mb genome while a reference-guided approach, yielded 843 Mb of genome sequence. The estimated GC content was 34.10%. Variant analysis revealed that BARI Kanthal-3 included 5.7 M (35%) and 10.4 M (65%) simple and heterozygous single nucleotide polymorphisms (SNPs), and about 90% of all these polymorphisms are in inter-genic regions. Through BUSCO assessment, 97.2% of the core genes were represented in the assembly with 1.3% and 1.5% either fragmented or missing, respectively. By comparing identified orthologous gene groups in BARI Kanthal-3 with five closely and one distantly related species of 10,092 common orthogroups were found across the genomes of the six species. The phylogenetic analysis of the shared orthogroups showed that *A. heterophyllus* was the closest species to BARI Kanthal-3 and orthogroups related to flowering time were found to be more highly prevalent in BARI Kanthal-3 compared to the other *Arctocarpus* spp. The findings of this study will help better understanding the evolution, domestication, phylogenetic relationships, year-round fruiting of this highly nutritious fruit crop as well as providing a resource for molecular breeding.

## 1 Introduction

Jackfruit (*Artocarpus heterophyllus* Lam), which belongs to the *Moraceae* family, has attracted the attention of food experts and technologists due to its nutritional health benefits (Zhang et al., 2022). It is a tropical evergreen tree, which produces the largest edible single fruit in the world (up to 50 kg/fruit) (Naik, 1949; Simmonds and Preedy, 2015; Lin et al., 2022). The place of origin of this fruit tree is still unclear, but it is widely grown in tropical countries including China, India, Malaysia, Thailand, Indonesia, Philippines and Bangladesh (Zhang et al., 2021b), and parts of central and eastern Africa, Florida (USA), Latin America and the Caribbean (Rahman et al., 1999; Sidhu, 2012; Sahu et al., 2020). Jackfruit (popularly known as ‘*Kanthal’*) is the national fruit of Bangladesh. Its demand is increasing gradually due to its low price, high nutritious value, diversified uses and potential for commercial cultivation (Sidhu, 2012). The crop is commonly referred to as “poor man’s food” due to its lower market price as well as high abundance in the summer season (Rahman et al., 1995; Sahu et al., 2020). Jackfruit flesh, the main edible portion, has a unique aroma and contains high levels of sugars (mainly sucrose, fructose and glucose), carboxylic acids, minerals, vitamins, and dietary fiber (Ong et al., 2006; Lin et al., 2022). The flesh is used as an ingredient in salads, or made into ice cream, jams, nectars, fruit bars, juices, chutney, cakes, jelly and fermented beverages (Zhang et al., 2021a). Notably, jackfruit seeds are rich in starch (60–80% based on dry matter), protein, vitamins and minerals, which may be boiled or roasted and eaten, or boiled and preserved in syrup like chestnuts (Anaya-Esparza et al., 2018).

Bangladesh is one of the largest producers of jackfruit and accounts for about 21% of total fruit production of the country, second only to Mango as the principal fruit crop. During 2019-20, Bangladesh produced 1.1 million tons of jackfruit covering 16,592 hectares area (Statistics., 2020). Despite its numerous advantages, jackfruit trees are not commercially grown as a crop because of an extremely high variation in fruit quality, which is due to its cross-pollinated nature, seed-mediated propagation, short seasonal fruiting and susceptibility to abiotic stresses (Sidhu, 2012). Therefore, the potential of this unique nutritious fruit crop has not yet been utilized in Bangladesh for ensuring food and nutritional security through commercial cultivation and industrial processing. Genetic improvement of existing germplasm to overcome these problems will accelerate jackfruit to become a commercial crop in Bangladesh (Dhar, 1998). A series of earlier studies evaluated the yield, quality and genetic diversity of jackfruit of Bangladesh but none of these studies are systematic and comprehensive (Hossain, 1996; Saha et al., 1996; Rahman et al., 2016) and the underlying molecular mechanisms of the trait diversity in jackfruit is largely unknown. The harvesting period of jackfruit is short (June-August) resulting in a large wastage of this fruit amounting to 20-30% of the crop or even more during some seasons. The Bangladesh Agricultural Research Institute (BARI) developed a year-round jackfruit variety namely, BARI Kanthal-3 in 2014. The number of fruits per plant per year ranges between 219-245 (average = 232), fruits are medium in size (averaging 5.43 kg each) and yield is 1,334.6 (ranges between 1165-1504.2) kg fruit/plant (**Table 1**). The ripened edible portion contains 35.06 mg/g ß- carotene and 23.6% of total soluble sugar (TSS) (Azad et al., 2007).

**Table 1:** Tree and fruit characteristics of *Artocarpus heterophyllus* L. (Year-round vs seasonal jackfruit).

Parameters	Features/traits		Reference (Seasonal jackfruit)
	Year-round (BARI Kanthal-3)	Seasonal jackfruit	
Average No. of fruits/plant	232	57	(Rahman et al., 2016)
Average fruit yield/plant/year (kg)	1,334	308	(Rahman et al., 2016)
Average fruit weight (kg)	5.43	5.8	(Rahman et al., 2016)
Harvesting period	September-June	June-August	(Goswami and Chacrabati, 2016)
Edible portion (%)	52.5	53.43	(Rahman et al., 2016)
pH	5.21	6.0	(Goswami and Chacrabati, 2016)
TSS (%)	23.6	19.87	(Rahman et al., 2016)
Vitamin C (mg/100g)	2.0	6.46	(Goswami and Chacrabati, 2016)
Total sugar (%)	21.74	14.95	(Goswami and Chacrabati, 2016)
Reducing sugar (%)	6.02	6.43	(Goswami and Chacrabati, 2016)
Nonreducing sugar (%)	15.72	8.52	(Goswami and Chacrabati, 2016)
Moisture content (%)	69.10	81.6	(Goswami and Chacrabati, 2016)
Dry matter	30.90	18.4	(Goswami and Chacrabati, 2016)

Whole-genome sequencing (WGS) provides complete coverage of the coding and noncoding regions of the genome (Galperin and Koonin, 2010), which allows a comprehensive assessment of the genome of any organism including those of plants (Chen et al., 2019). It provides a genetic foundation that enables a greater efficiency to identify genetic diversity at key genes that can be used to enhance, reduce or add certain features to a plant phenotype (Chen et al., 2019). Since the first WGS of the model plant, *Arabidopsis thaliana* in 2000, a large number of plants from diverse taxonomic groups have been sequenced, and genes responsible for various plant traits have been characterized and cloned (Gardner et al., 2016; Chen et al., 2019; Hübner et al., 2019). Recently, WGS of economically important plants and animals such as jute, *Corchorus* spp. (Islam et al., 2017), hilsa (*Tenualosa ilisha*) (Das et al., 2018), and goat (*Capra hircus*) (Siddiki et al., 2019) have created huge public interest in Bangladesh. Recent advances in genomic analyses have revealed large numbers of single nucleotide polymorphisms (SNPs) as the most common form of DNA sequence variation between alleles in several plant species (Morgil et al., 2020). Because of their high abundance, significant information content, when associated with genes, SNPs have gained the center stage as the principal markers of choice for molecular genetics studies. This includes their application in shortening the time of breeding new varieties in many crops through marker assisted selection (Mammadov et al., 2012; Morgil et al., 2020). SNPs have also been applied for several years to assess diversity in specific genes or genomic regions, revealing the phylogenetic relationships between species. However, the emergence of high throughput sequencing technologies allows the SNP-based genetic diversity studies to be carried out at scale and can be useful in conserving diversity in domesticated populations. Plant phylogenetic and evolutionary studies are conventionally based on variation that exist at genes, and hence the knowledge of SNPs in these regions is essential for this analysis (Lasky et al., 2012). It is also important to know the location of SNPs in the whole genome, because if a SNP is present in the coding or regulatory region of a gene, it can greatly affect the functional activity of the resulting protein, such as an enzyme in a biosynthetic pathway (Somerville and Koornneef, 2002) by affecting gene expression and transcriptional and translational promoter activities. Therefore, SNPs can often be responsible for phenotypic variations that exits between individuals and be utilized as selectable genetic markers for improving agronomic traits.

However until now, only a limited amount of genomic information has been made available for the genus of *A. heterophyllus* (Laricchia et al., 2018; Sahu et al., 2020; Lin et al., 2022). Although the development of a year-round fruiting variety BARI Kanthal-3 from a wild accession offers an opportunity for commercial cultivation and processing of the jackfruit, nothing is known about the underlying molecular mechanism of its year-round fruiting characteristics and other beneficial traits. Molecular understanding of the extremely high phenotypic variabilities in jackfruit would facilitate the future development of high yielding, year-round fruiting, biotic and abiotic stress (e.g., flood, saline, drought, pathogen and pest) tolerant jackfruit varieties through molecular breeding, which is essential for establishing a jackfruit-based processing industry in Bangladesh and elsewhere. We report here an annotated Whole-genome assembly of the year-round fruiting *A. heterophyllus* cv. BARI Kanthal-3 for first time. The results of the promising phenotypic characteristics of the BARI Kanthal-3 variety, together with both the *de novo* and reference-guided assemblies, the identified SNPs, and copy number variation in genes that impact flowering time sheds light on the genetic diversity that exists within the *A. heterophyllus* genome.

## 2 Materials and methods

### 2.1 Collection of phenotypic data

The mean values (n = 5) of the phenotypic and biochemical data on tree and fruit characteristics of *Artocarpus heterophyllus* (BARI Kanthal-3) were obtained using the standard protocols, and compared with the published data of seasonal jackfruit (Goswami and Chacrabati, 2016; Rahman et al., 2016).

### 2.2 Collection of leaf samples, genomic DNA extraction, WGS library construction, and sequencing

The year-round jackfruit cultivar ‘BARI Kanthal-3’, a superior variety of jackfruit of Bangladesh, was used for genome sequencing. We collected fresh leaf samples from germplasm repository at BARI, Joydebpur, Bangladesh. The samples were identified as jackfruit by Professor Md. Abdul Baset Mia of Department of Crop Botany of Bangabandhu Sheikh Mujibur Rahman Agricultural University (BSMRAU) in Bangladesh. A voucher specimen was deposited in the herbarium of the Department of Crop Botany of BSMRAU with an accession No. VS (HM) 005/2021. The origin of this germplasm is from a wild accession found in Chattogram Hiltracts (Ramgarh) of Bangladesh. Genomic DNA was extracted from freshly harvested leaves using the QIAGEN DNeasy Plant Mini Kit (QIAGEN, Valencia, California, USA) following the manufacturer’s protocol. The quality of the DNA was visually inspected by 1% agarose gel electrophoresis. The quantity of the DNA was assessed by a Qubit Fluorometer (Invitrogen, Carlsbad, CA, USA) according to the manufacturer’s instruction. Whole-genome sequencing (WGS) library preparation was performed using Nextera XT DNA library preparation kit (Illumina Inc., San Diego, CA, USA) according to the manufacturer’s protocol. Briefly, after normalization, DNA samples were fragmented and tagged by tagmentation in a single-tube reaction (Hoque et al., 2019). The tagmented DNA was amplified through a limited-cycle PCR program using a unique combination of barcode primers, the Index 1 (i7), Index 2 (i5) and full adapter sequences required for cluster generation. Amplification was followed by a cleanup step that purified the library DNA, and removed small library fragments by using Agencourt AMPure XP beads (Beckman Coulter, Inc.). Finally, prepared libraries were loaded onto a reagent cartridge, clustered on the NextSeq 550 System, and paired-end sequencing (2×150 bp) was performed using the Illumina NextSeq 550 High-Output Kit on the NextSeq 550 desktop sequencer.

### 2.3 Retrieval of data from the GenBank

In addition to the WGS data of BARI Kanthal-3, we also collected the genomic data (WGS) of five related and one distantly related species viz., *Artocarpus heterophyllus* (family *Moraceae*) i.e., *A. heterophyllus, A. altilis, Morus notabilis, Arabidopsis thaliana* and *Ficus carica* from the AOCC ORCAE platform and the National Center for Biotechnology Information (NCBI) under GenBank accession numbers of CNGB CNP0000486 (https://bioinformatics.psb.ugent.be/orcae/aocc/overview/Arthe), CNGB CNP0000715 (https://bioinformatics.psb.ugent.be/orcae/aocc/overview/Artal), NCBI ASM41409v2 (https://www.ncbi.nlm.nih.gov/genome/?term=ASM41409v2), NCBI TAIR10.1 (https://www.ncbi.nlm.nih.gov/genome/?term=TAIR10.1) and, NCBI Bioproject PRJNA565858, respectively.

### 2.4 *De novo* genome assembly and annotation

The generated WGS data were filtered through Trimmomatic v0.38 (Bolger et al., 2014) with option “LEADING:20 TRAILING:20 SLIDINGWINDOW:4:15 MINLEN:50” parameters to remove Illumina adapter, known Illumina artifacts, phiX, and low-quality regions. The processed reads were assembled by SOAPdenovo2 v2.04 (Luo et al., 2012) with k-mer=39 and subsequently scaffolded using a reference guided approach by RAGTAG (Alonge et al., 2019) software with default parameters. GapCloser v1.12 (Luo et al., 2012) with default parameters (“-l 150 -t 32 -p 31”) was utilized for gap closing using the pair-end data. The assembled genome was analyzed by EDTA (Ou et al., 2019) software to create a non-redundant transposable element database which was then used to soft-mask the genome sequence. Genome annotation was performed by using Braker2 (Brůna et al., 2021) software with coding-gene sequence evidence from *A. heterophyllus*.

### 2.5 Genome assembly validation

The scaffolded sequences were compared against the reference genome by nucmer v4.0.0rc1 (Kurtz et al., 2004) using the default parameters. The genome assembly completeness was assessed using BUSCO (Benchmarking Universal Single-Copy Orthologues), v4.1.4 (Seppey et al., 2019) to evaluate the presence of conserved plant orthologs with the Embryophyta database 10 lineage.

### 2.6 Genome size estimation

The high-quality data (~50X depth) were provided to Jellyfish v2.2.6 (Marçais and Kingsford, 2011) with “-C -m 21 -s 5G –min-quality=25” parameters to generate k-mer (K=21) frequency distribution. The output histograms were analyzed using GenomeScope (Vurture et al., 2017) to estimate the genome size, heterozygosity level, error rates, and repeat fraction.

### 2.7 Variant calling

Processed Illumina data were aligned against the draft *A. heterophyllus* genome (Sahu et al., 2020) using Burrows-Wheeler Aligner (BWA) v 0.7.17 (Li and Durbin, 2009). The aligned reads mapped with a quality score 30 or greater were analyzed by samtools/bcftools (Li, 2011) to produce the raw variant calls. Simple single nucleotide polymorphisms (SNPs) were defined as SNPs with greater than 90% ALT allele frequency observed in at least 30 high quality reads (MAPQ>30). Following variant calling, we used snpEff to annotate variants and predict their effects on genes using a custom database generated using the *A. heterophylus* genome and annotation from https://bioinformatics.psb.ugent.be/gdb/aocc/arthe/ (Sahu et al., 2020).

### 2.8 Orthologous gene analysis

Gene orthology and orthogroups were inferred by Orthofinder (Emms and Kelly, 2019) based on protein sequence similarity searches using Diamond (Buchfink et al., 2015) software. 306 genes involved in *A. thaliana* flowering-time gene networks were downloaded from the Flowering-Interactive Database (FLOR-ID) (Bouché et al., 2016). Gene expansion or contraction were assessed based on relative gene count within each orthogroup relative to *A. thaliana*.

## 3 Results and discussion

### 3.1 Source and phenotypic features of the BARI Kanthal-3 variety

**Figure 1:**
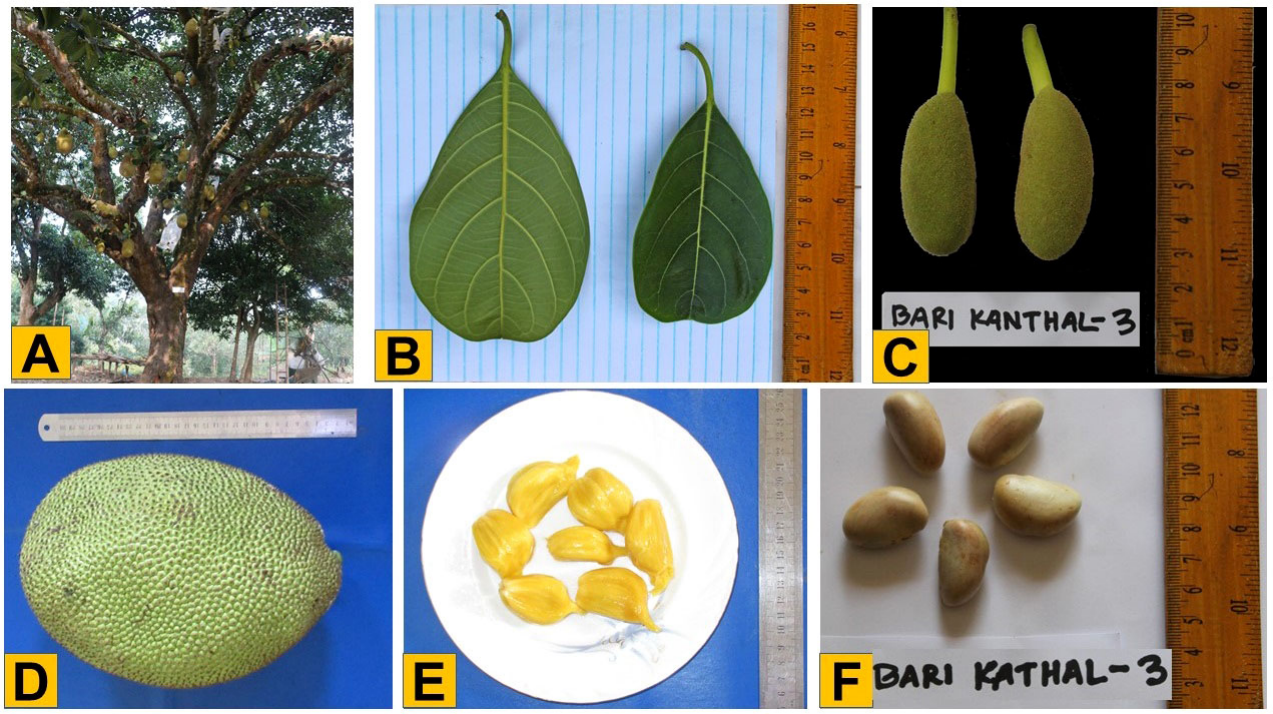
Phenotypic characteristics of BARI Kanthal-3. **(A)** Tree *in situ*, **(B)** Leaf, **(C)** Young fruits from the tree, **(D)** Mature fruit, **(E)** Kernel (flesh), and **(F)** Seed.

To develop the variety of BARI Kanthal-3, germplasm was collected from locations all over the Bangladesh including in the Chattogram Hiltracts, such as Ramgarh of Khagrachari. In 2014, an accession of Ramgarh was certified for cultivation in Bangladesh with a varietal name of BARI Kanthal-3, representing a new and unique variety of jackfruit in Bangladesh that bear fruits for ten months of the calendar year (September to June) while the seasonal plant gives fruit only for three months (June to August) each year. This year-round fruiting variety produces more than 4-fold higher average number of fruits per plant and fruit yield per plant per year compared to seasonal jackfruit. A mature plant produces an average of 232 (range 219-245) fruits per plant yielding about 1,334.6 kg (1165 -1504.2 kg) fruit/plant/year (**Table 1**). The fruits of BARI Kanthal-3 were medium (average 5.43 kg each) and average yield was 133.2 t/ha/year. This variety was not affected by any sort of infectious pathogens or pests (data not shown). The tree is erect and medium bushy, and the pulp of the fruit is medium soft, slightly yellow, medium juicy, highly sweet and aromatic (**Figure 1**). The amounts of ß- carotene and total soluble solid (TSS) in fruits were 35.06 mg/g and 23.6%, respectively. The edible portion of the fruit was 52.5% (**Table 1**). A large body of literature has revealed that jackfruit is a rich source of carbohydrates, minerals, carboxylic acids, dietary fiber, vitamins and minerals and bioactive compounds (Azad et al., 2007; Khan et al., 2021). Clearly, BARI Kanthal-3 is an extremely high yielding (ca. 4-fold higher than average fruit yield of a seasonal jackfruit per year) and high quality jackfruit. 

### 3.2 Genome sequencing, assembly and annotation

**Figure 2:**
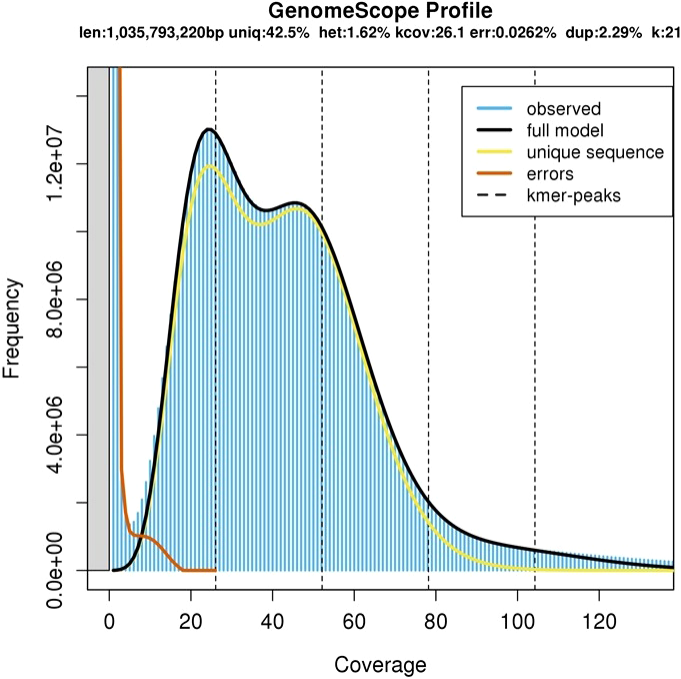
Genome size prediction of *Artocarpus heterophyllus* cv. BARI Kanthal-3. The X-axis represents the coverage of the genome while the Y-axis represents the frequency levels.

A total of 472 million raw Illumina pair-end sequencing reads from the extracted DNA of *A. heterophyllus* cv. BARI Kanthal-3 leaf tissues were generated. The genome size of BARI Kanthal-3 was estimated to be 1.04 Gb with a heterozygosity rate of 1.62% based on K-mer analysis of the short read data (**Figure 2**). The estimated size is similar to the recently reported 1.01Gb genome size of seasonal *A. heterophyllus* (Sahu et al., 2020), and is consistent with the c-value of 1.20 pg (Ohri and Kumar, 1986). BARI Kanthal-3 has a higher heterozygosity rate compared to the available reference genomes of seasonal jackfruit recently published, at 0.90 and 0.91, respectively (Sahu et al., 2020; Lin et al., 2022).

After quality filtering of the short reads using Trimmomatic, 439 million clean reads were obtained (**Table 2**). The high-quality reads assembled into different contigs using SOAPdenovo2, which ultimately yielded a base assembly of 1.36 M scaffolds, totaling 817.7 Mb. The N50s of scaffolds were 1.8 Kb (**Table 2**). The BARI Kanthal-3 contigs were then further scaffolded together using the reference guided approach using the existing published draft reference genome of jackfruit (Sahu et al., 2020) and the software RAGTAG, and finally gaps in the scaffolds were filled using the same pair-end Illumina data and Gapcloser software. In this case, SOAPdenovo2 + RAGTAG + GapCloser produced a base assembly of N50 size = 425 Kb in 218,562 scaffolds (**Table 2**). The mapping of paired-end short read data against the *de novo* scaffolded assembly and out of 439 million read pairs, 400 million (91.1%) aligned to the assembly were properly paired indicating a high proportion of the original data is represented in the assembly. The GC content of BARI Kanthal-3 was 34.10% which is comparable to the GC content of a seasonal *A. heterophyllus* from Indian and Chinese origins that were recorded at 32.9% and 34.9%, respectively (**Table S1**) (Sahu et al., 2020; Lin et al., 2022).

**Table 2:** Result of *de novo* and reference guided genome assembly of *Artocarpus heterophyllus* cv. BARI Kanthal-3.

Assembly	N50 number	N50 size (Kb)	N90 number	N90 size (Kb)	Longest Sequence (Kb)	Num Sequence	Total Bases (Mb)
SOAPdenovo2	88,402	1.8	720,032	0.2	65	1,364,052	817.7
SOAPdenovo2 + RAGTAG + Gapcloser	561	425	14,076	1.4	2,611	218,562	843

We annotated 41,088 protein-coding genes in BARI-Kanthal-3 assembly using the Braker2 gene prediction pipeline. The gene number is comparable to the two existing *A. heterophyllus* genome assemblies with 35,858 (Sahu et al., 2020) and 41,997 (Lin et al., 2022) annotated genes, respectively.

### 3.3 Genome assembly validation

**Figure 3:**
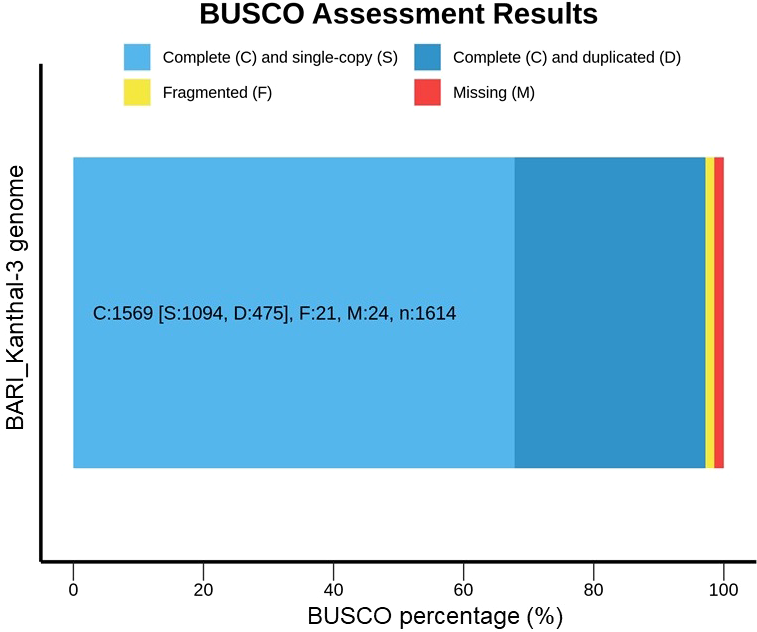
BUSCO (v.4.0.4) assessment results of BARI Kanthal-3. BUSCO completeness in *Artocarpus heterophyllus* cv. BARI Kanthal-3 is high indicating that the genome assemblies are of high gene space completeness.

To assess the representation of a complete conserved core gene set in the BARI Kanthal-3, assembly, an analysis was carried out to assess the quality and completeness of the draft genome using the Benchmarking Universal Single-Copy Orthologs (BUSCO) datasets and an orthologue data base (**Figure 3**). We identified 1,614 single copy orthologs (SCOs). Among these SCOs, 97.2% (1569/1614) were complete (single copy = 1094, and duplicated = 475), whereas 21 and 24 were fragmented and missing, respectively (**Figure 3**). Our present findings aligned with the recently reported findings of Sahu et al. (2020), who reported that out of 1440 BUSCO ortholog groups searched in the *A. heterophyllus* assembly, 95% (1369/1440) were complete BUSCOs, 932 (64.7%) were “complete single-copy”, 437 (30.3%) were “complete duplicated”, and 15 (1%) were “fragmented”, and 56 (4%) were “missing”.

**Figure 4:**
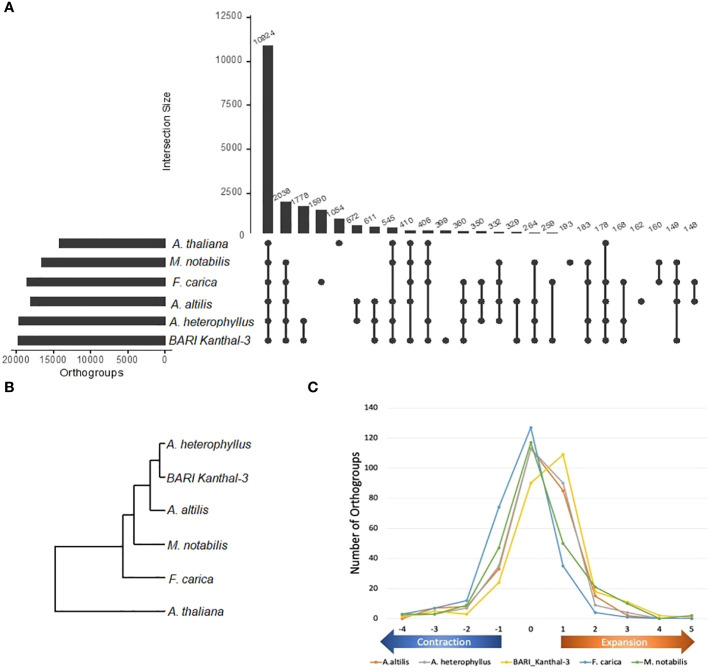
Distribution of orthogroups in the genomes of five species (*Artocarpus heterophyllus*, *A. altilis*, *Ficus carica*, *Morus notabilis*, *Arabidopsis thaliana* and BARI Kanthal-3). **(A)** diagram showing unique and shared orthogroups in three genomes of *Artocarpus* species, *M. notabilis*, *F. carica* and *Arabidopsis*. Each number represents the number of orthogroups in common between each pairing. **(B)** Inferred phylogenetic tree constructed with common orthogroups from *A. heterophyllus*, *A. altilis*, *M. notabilis*, *F. carica*, *A. thaliana* and BARI Kanthal-3 (*A. heterophyllus)* genomes. The tree was constructed using rooted gene tree method in OrthoFinder. **(C)** Relative numbers of flowering gene orthogroups in each of the Moraceae genomes compared to *Arabidopsis thaliana*.

We then carried out an orthologous gene analysis across the BARI Kanthal-3 genome and the genomes of five other species (*A. heterophyllus*, *A. altilis*, *F. carica*, *M. notabilis*, *A. thaliana*) based upon a protein similarity search using Diamond software and the inference of gene orthology and orthogroups using Orthofinder software. In this study, 10,924 orthogroups were found to be shared across the genomes of the six species (**Figure 4A**) with 399 found to be unique for BARI Kanthal-3 genome. Phylogenetic analysis was performed across the 10,924 shared orthogroups using the rooted gene tree method in Orthofinder (**Figure 4B**). The analysis revealed that the two genomes of *A. heterophylus* (BARI Kanthal-3 and *A. heterophyllus*) clustered more closely related to the other three *Moraceae* genomes with *A. thaliana* as an outgroup in the phylogenetic tree (**Figure 4B**). Finally, to assess if there was a relationship between differences in flowering time between BARI Kanthal-3 and seasonal *A. heterophyllus* and differences in the representation of orthogroups related to flowering time between them, we identified the relative numbers of these orthogroups in each of the *Moraceae* genomes compared to Arabidopsis (**Figure 4C** and **Table S2**). This identified large scale expansion in the *Artocarpus* spp. likely reflecting the whole genome duplication event previously identified in their evolution (Lin et al., 2022). However, a higher number of duplications were identified in the BARI Kanthal-3 genome compared to the other *Artocarpus* spp. indicating the longer season nature of the accession could be explained by an overall expansion of these gene families, although the more fractured nature of the genome assembly could also be impacting this observation. The availability of more contiguous long read genome assemblies for BARI Kanthal-3 and other *Artocarpus* spp. would enable this observation to be confirmed.

### 3.4 Variant analysis

**Figure 5:**
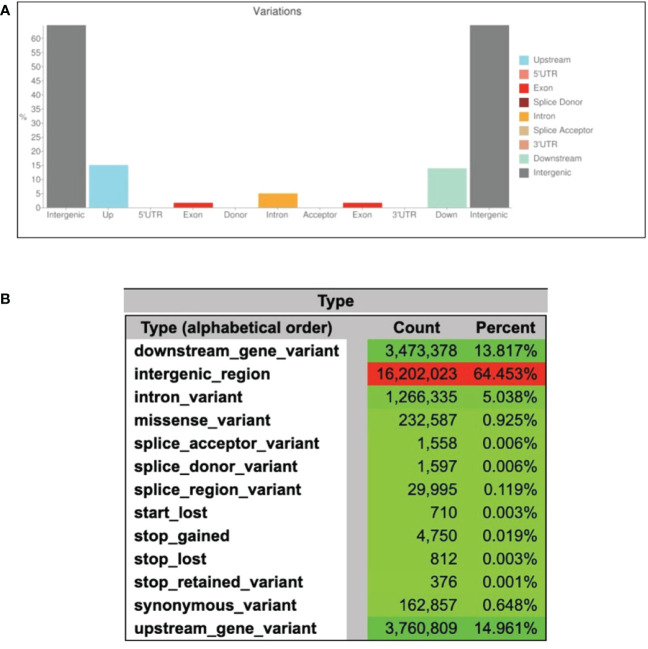
Variant annotation and effects of variant on genes. **(A)** Distribution and abundance of variants in the genome of BARI Kanthal-3, and **(B)** Number of effects by type and region of the gene variants.

The processed WGS reads were aligned against the *A. heterophyllus* draft assembly. Out of a total of 439 M reads, 417 M (95.1%) were found to be aligned in exact pairs. A total of 16 million single-nucleotide polymorphisms (SNPs) were called from the dataset including 5.7 M (35.0%) simple and 10.4 M (65.0%) heterozygous SNPs (**Table 3** and **Figure S1**). Approximately, 90% of all polymorphisms are located in intergenic regions. In this study, 144,787 (2.5%) and 426,997 (7.5%) of the simple SNPs, and 250,715 (2.4%) and 739,288 (7.1%) of the heterozygous SNPs, were found in the exons and introns, respectively (**Table 3**). We further predicted the effects of variants on genes. As expected, large fraction of the variants was in the intergenic (64.5%), intronic (5%) and up/down-stream regions (29%) of the genes (**Figure 5A**). There are 232,587 missense mutations and 4,750 gained stop codons suggesting an altered protein function in BARI Kanthal-3 (**Figure 5B**). One of the important findings of this study is the high level of heterozygosity in the year-round fruiting jackfruit genome. The high level of heterozygosity in *A. heterophyllus* genome raises the question of which allele, for each heterozygous locus, is represented by the reference genome (**Table S2**). Therefore, the inherent differences between individual plants should always be considered when utilizing the reference genome to detect SNP variants (Hawkins et al., 2016).

**Table 3:** Results of variant analysis of *Artocarpus heterophyllus* cv. BARI Kanthal-3.

Genomic region	Simple SNPs	Heterozygous SNPs
Exonic	144,787	250,715
Intronic	426,997	739,288
Inter-genic	5,111,742	9,369,507
Total	5,683,526	10,359,510

SNPs have been indicated as the major factors in the creation of phenotypic variation and their effect on functional changes of genes is used as a tool in functional genomics of organisms (Hirakawa et al., 2013). In plants, many traits of interest have been linked with SNPs (Mammadov et al., 2012; Huq et al., 2016; Zhang et al., 2018), including roles in metabolism, cellular processes and signaling, that could have an impact on breeding. This study for the first time identified the SNPs in the genome of a year-round fruiting jackfruit cultivar, which promises the development of genetic markers associated with the important traits of this economically important species including the genes regulating flowering and fruit development. The availability of SNPs within the coding and regulatory sequences also offers the prospect of identifying the causative variations influencing these processes (Varshney, 2010). One of the hallmark findings of this study is that majority of the SNPs (47.29%) of BARI Kanthal-3 were localized in the intergenic regions in the proximity of genes (including 5′ UTR, 3′ UTR, and introns). Approximately, 25% of the intergenic SNPs were detected within the region spanning 10 kb upstream of the gene start site and 10 kb downstream of the gene end site (Yamamoto et al., 2007), implying the possibility that some of these SNPs affect the expression of the nearest neighboring genes. It has been reported that a high frequency of genetic variants in the noncoding regions likely results from less selection pressure from natural selection and/or domestication (Barreiro et al., 2008). However, DNA polymorphisms in these regions have been reported to play important roles during evolution and domestication. For example, a mutation in the 5′ regulatory region of the *qSH1* gene, an ortholog of the *Arabidopsis* homeobox gene *REPLUMLESS* (*RPL*) results in the absence of abscission zone formation and thus loss of seed shattering in a subset of temperate *japonica* cultivar of rice (Konishi et al., 2006). Similarly, a considerable number of mutations in introns in pre-harvest sprouting (PHS) genes lead to PHS in rice plant (Lee et al., 2017). Among the 12 PHS mutants (phs), mutations in genes encoding major enzymes of the carotenoid biosynthesis pathway, cause photo-oxidation and ABA-deficiency phenotypes, of which the latter is a major factor controlling the PHS trait in rice (Fang et al., 2008). Interestingly, in jackfruit, MADS-box genes and carotenoid biosynthesis genes, were the primary targets for domestication (Laricchia et al., 2018). However, the role of inter-genic SNPs in *A. heterophylus* in domestication of this horticultural plant needs to be explored further.

SNP markers have become extremely popular in plant molecular genetics due to their genome-wide abundance and amenability for high-throughput detection platforms. For example, SNPs regulating various Quantitative Trait Loci (QTL) responsible for cold and disease resistance such as such as blight, bacterial canker and gray mold have been reported (Zhang et al., 2003; Coaker and Francis, 2004), and SNPs associated with flowering in *Raphanus sativus* have recently been discovered by transcriptome sequencing and computational analysis (Kim et al., 2019). An additional transcriptomics study in BARI Kanthal-3 is needed for the identification of genes associated with flowering time and year-round fruiting.

## 4 Conclusions

Although genomic information has been made available for seasonal jackfruit, this is the first report of an assembled and annotated genome for a year-round fruiting jackfruit variety. The annotation yielded a very similar number of annotated genes to a recent genome assembly of a seasonal jackfruit together with a high representation of core othologous genes. An orthologous gene analysis with other Moraceae gennomes confirmed the close realtionship between BARI Kanthal-3 and other *Arctocarpus* spp. but also indicated a higher number of genes related to flowering time were present in the year-round fruiting variety.

This study also reported the distribution of a large collection of nucleotide variation identified across the genome that can be used to identify new functional genes and their regulatory activities specific to BARI Kanthal-3. Furthermore, the genomic data and the identified SNPs of this year-round fruiting jackfruit cultivar could facilitate further genomics and post-genomics studies for detecting other trait-specific genes that are essential for molecular breeding of jackfruit. One of the limitations of this study is lack of information on the role of inter-genic SNPs in BARI Kanthal-3 and the impact on gene function (i.e., changes in protein-coding genes, differential gene expression, and the specific functions of protein-coding genes). To further understand the underlying molecular mechanisms of unique traits of this new variety, a high quality long read genome assembly of BARI-Kanthal-3 and comparative transcriptome analysis with seasonal jackfruit are needed.

The fruit nutritional quality, yield and year-round fruiting properties of BARI Kanthal-3 indicate a unique and valuable genetic material for the improvement of commercial cultivation and development of jackfruit-based processing industry in Bangladesh. The genomic data, associated orthogroups, and SNPs identified in this research will be useful for characterization of trait-specific genes and development of markers for molecular breeding for the improvement of jackfruit, and provides an opportunity to develop this underutilized crop for ensuring food and nutritional security for the increasing population of Bangladesh and other tropical countries.

## Data availability statement

The datasets presented in this study can be found in online repositories. The names of the repository/repositories and accession number(s) can be found at https://www.ncbi.nlm.nih.gov/nuccore/JAIQDR000000000.1, https://www.ncbi.nlm.nih.gov/bioproject/PRJNA686208.

## Ethics statement

Permission to collect leaf sample of *Artocarpus heteropyllus* Lam. was obtained.

## Author contributions

All authors contributed intellectually to this study. TI conceived the study, designed the experiment, coordinated the project, provided reagents and laboratory support, interpreted the results, wrote and revised the manuscript. NA collected the plant samples, extracted DNA, and prepared the draft manuscript; CK assembled and annotated the sequenced data, interpreted results and wrote the manuscript; MNH, interpreted the results, wrote and revised the manuscript; MJH, provided plant samples, collected and interpreted phenotypic and biochemical data and wrote manuscript; NUM and DRG, conducted experiments and prepared library for the DNA sequencing using Illumina Nextseq 550; AAN and RI, analyzed sequenced data, prepared phylogenetic tree and wrote the manuscript; PKB, wrote and revised the manuscript; AGS, coordinated, wrote and revised the manuscript; All authors read, revised, edited and approved the final manuscript.

## Supplementary material

Supplementary Table 1 Comparison between the previously reported *Artocarpus heterophyllus* sequence and BARI Kanthal-3 sequence.

Supplementary Table 2 Flowering locus C (FLC). Listing of flowering gene orthologues (Enclosed in a separate file).

Supplementary Figure 1 Categories of candidate SNPs identified in the genome of BARI_Kanthal-3.

## References

[B1] Alonge M. Soyk S. Ramakrishnan S. Wang X. Goodwin S. Sedlazeck F. J.. (2019). RaGOO: fast and accurate reference-guided scaffolding of draft genomes. Genome Biol. 20, 1–17. doi: 10.1186/s13059-019-1829-6 31661016 PMC6816165

[B2] Anaya-Esparza L. M. González-Aguilar G. A. Domínguez-Ávila J. A. Olmos-Cornejo J. E. Pérez-Larios A. Montalvo-González E. (2018). Effects of minimal processing technologies on jackfruit (Artocarpus heterophyllus lam.) quality parameters. Food Bioprocess. Technol. 11, 1761–1774. doi: 10.1007/s11947-018-2136-z

[B3] Azad A. Jones J. Haq N. (2007). Assessing morphological and isozyme variation of jackfruit (Artocarpus heterophyllus lam.) in Bangladesh. Agroforestry Syst. 71, 109–125. doi: 10.1007/s10457-007-9039-8

[B4] Barreiro L. B. Laval G. Quach H. Patin E. Quintana-Murci L. (2008). Natural selection has driven population differentiation in modern humans. Nat. Genet. 40, 340–345. doi: 10.1038/ng.78 18246066

[B5] Bolger A. M. Lohse M. Usadel B. (2014). Trimmomatic: a flexible trimmer for illumina sequence data. Bioinformatics 30, 2114–2120. doi: 10.1093/bioinformatics/btu170 24695404 PMC4103590

[B6] Bouché F. Lobet G. Tocquin P. Périlleux C. (2016). FLOR-ID: an interactive database of flowering-time gene networks in arabidopsis thaliana. Nucleic Acids Res. 44, D1167–D1171. doi: 10.1093/nar/gkv1054 26476447 PMC4702789

[B7] Brůna T. Hoff K. J. Lomsadze A. Stanke M. Borodovsky M. (2021). BRAKER2: automatic eukaryotic genome annotation with GeneMark-EP+ and AUGUSTUS supported by a protein database. NAR. Genomics Bioinf. 3, lqaa108. doi: 10.1093/nargab/lqaa108 PMC7787252 33575650

[B8] Buchfink B. Xie C. Huson D. H. (2015). Fast and sensitive protein alignment using DIAMOND. Nat. Methods 12, 59–60. doi: 10.1038/nmeth.3176 25402007

[B9] Chen F. Song Y. Li X. Chen J. Mo L. Zhang X.. (2019). Genome sequences of horticultural plants: past, present, and future. Horticult. Res. 6, 1–23. doi: 10.1038/s41438-019-0195-6 PMC6804536 31645966

[B10] Coaker G. Francis D. M. (2004). Mapping, genetic effects, and epistatic interaction of two bacterial canker resistance QTLs from lycopersicon hirsutum. Theor. Appl. Genet. 108, 1047–1055. doi: 10.1007/s00122-003-1531-6 15067391

[B11] Das A. Ianakiev P. Baten A. Nehleen R. Ehsan T. Ahmed O.. (2018). Genome of tenualosa ilisha from the river padma, Bangladesh. BMC Res. Notes 11, 1–3. doi: 10.1186/s13104-018-4028-8 30577879 PMC6303923

[B12] Dhar M. (1998). Techniques of vegetative and *in vitro* propagation of jackfruit, Salna, Gazipur Bangladesh:Institute Postgraduate Studies in Agriculture. Ph D Thesis, pp. 27–31

[B13] Emms D. M. Kelly S. (2019). OrthoFinder: phylogenetic orthology inference for comparative genomics. Genome Biol. 20, 1–14. doi: 10.1186/s13059-019-1832-y 31727128 PMC6857279

[B14] Fang J. Chai C. Qian Q. Li C. Tang J. Sun L.. (2008). Mutations of genes in synthesis of the carotenoid precursors of ABA lead to pre-harvest sprouting and photo-oxidation in rice. Plant J. 54, 177–189. doi: 10.1111/j.1365-313X.2008.03411.x 18208525 PMC2327239

[B15] Galperin M. Y. Koonin E. V. (2010). From complete genome sequence to ‘complete’understanding? Trends Biotechnol. 28, 398–406. doi: 10.1016/j.tibtech.2010.05.006 20647113 PMC3065831

[B16] Gardner E. M. Johnson M. G. Ragone D. Wickett N. J. Zerega N. J. (2016). Low-coverage, whole-genome sequencing of artocarpus camansi (Moraceae) for phylogenetic marker development and gene discovery. Appl. Plant Sci. 4, 1600017. doi: 10.3732/apps.1600017 PMC4948901 27437173

[B17] Goswami C. Chacrabati R. (2016). ““Jackfruit (*Artocarpus heterophylus*),,” in MSJ. Simmonds and V. R. Preedy (Eds) Nutritional composition of fruit cultivars (Elsevier), 317–335. doi: 10.1016/B978-0-12-408117-8.00014-3

[B18] Hawkins C. Caruana J. Schiksnis E. Liu Z. (2016). Genome-scale DNA variant analysis and functional validation of a SNP underlying yellow fruit color in wild strawberry. Sci. Rep. 6, 1–11. doi: 10.1038/srep29017 27377763 PMC4932534

[B19] Hirakawa H. Shirasawa K. Ohyama A. Fukuoka H. Aoki K. Rothan C.. (2013). Genome-wide SNP genotyping to infer the effects on gene functions in tomato. DNA Res. 20, 221–233. doi: 10.1093/dnares/dst005 23482505 PMC3686429

[B20] Hoque M. N. Istiaq A. Clement R. A. Sultana M. Crandall K. A. Siddiki A. Z.. (2019). Metagenomic deep sequencing reveals association of microbiome signature with functional biases in bovine mastitis. Sci. Rep. 9, 1–14. doi: 10.1038/s41598-019-49468-4 31537825 PMC6753130

[B21] Hossain A. (1996). Status report on genetic resources of jackfruit in Bangladesh (Singapore: International Plant Genetic Resources Institute Regional Office).

[B22] Hübner S. Bercovich N. Todesco M. Mandel J. R. Odenheimer J. Ziegler E.. (2019). Sunflower pan-genome analysis shows that hybridization altered gene content and disease resistance. Nat. Plants 5, 54–62. doi: 10.1038/s41477-018-0329-0 30598532

[B23] Huq M. A. Akter S. Nou I. S. Kim H. T. Jung Y. J. Kang K. K. (2016). Identification of functional SNPs in genes and their effects on plant phenotypes. J. Plant Biotechnol. 43, 1–11. doi: 10.5010/JPB.2016.43.1.1

[B24] Islam M. S. Saito J. A. Emdad E. M. Ahmed B. Islam M. M. Halim A.. (2017). Comparative genomics of two jute species and insight into fibre biogenesis. Nat. Plants 3, 1–7. doi: 10.1038/nplants.2016.223 28134914

[B25] Khan A. U. Ema I. J. Faruk M. Tarapder S. A. Khan A. U. Noreen S. (2021). A review on importance of artocarpus heterophyllus L. (Jackfruit) J. Multidiscip. Appl. Natural Science 1 (2), 106–116. doi: 10.47352/jmans.v1i2.88

[B26] Kim J. Manivannan A. Kim D.-S. Lee E.-S. Lee H.-E. (2019). Transcriptome sequencing assisted discovery and computational analysis of novel SNPs associated with flowering in raphanus sativus in-bred lines for marker-assisted backcross breeding. Horticult. Res. 6, 1–12. doi: 10.1038/s41438-019-0200-0 PMC6823433 31700647

[B27] Konishi S. Izawa T. Lin S. Y. Ebana K. Fukuta Y. Sasaki T.. (2006). An SNP caused loss of seed shattering during rice domestication. Science 312, 1392–1396. doi: 10.1126/science.1126410 16614172

[B28] Kurtz S. Phillippy A. Delcher A. L. Smoot M. Shumway M. Antonescu C.. (2004). Versatile and open software for comparing large genomes. Genome Biol. 5, 1–9. doi: 10.1186/gb-2004-5-2-r12 PMC395750 14759262

[B29] Laricchia K. M. Johnson M. G. Ragone D. Williams E. W. Zerega N. J. Wickett N. J. (2018). A transcriptome screen for positive selection in domesticated breadfruit and its wild relatives (Artocarpus spp.). Am. J. Bot. 105, 915–926. doi: 10.1002/ajb2.1095 29882953

[B30] Lasky J. R. Des Marais D. L. Mckay J. K. Richards J. H. Juenger T. E. Keitt T. H. (2012). Characterizing genomic variation of arabidopsis thaliana: the roles of geography and climate. Mol. Ecol. 21, 5512–5529. doi: 10.1111/j.1365-294X.2012.05709.x 22857709

[B31] Lee G.-A. Jeon Y.-A. Lee H.-S. Hyun D. Y. Lee J.-R. Lee M.-C.. (2017). New genetic loci associated with preharvest sprouting and its evaluation based on the model equation in rice. Front. Plant Sci. 8, 1393. doi: 10.3389/fpls.2017.01393 28848592 PMC5550670

[B32] Li H. (2011). A statistical framework for SNP calling, mutation discovery, association mapping and population genetical parameter estimation from sequencing data. Bioinformatics 27, 2987–2993. doi: 10.1093/bioinformatics/btr509 21903627 PMC3198575

[B33] Li H. Durbin R. (2009). Fast and accurate short read alignment with burrows–wheeler transform. bioinformatics 25, 1754–1760. doi: 10.1093/bioinformatics/btp324 19451168 PMC2705234

[B34] Lin X. Feng C. Lin T. Harris A. Li Y. Kang M. (2022). Jackfruit genome and population genomics provide insights into fruit evolution and domestication history in China. Horticult. Res. 9, 1–13. doi: 10.1093/hr/uhac173 PMC9533223 36204202

[B35] Luo R. Liu B. Xie Y. Li Z. Huang W. Yuan J.. (2012). SOAPdenovo2: an empirically improved memory-efficient short-read *de novo* assembler. Gigascience 1, 18. doi: 10.1186/2047-217X-1-18 23587118 PMC3626529

[B36] Mammadov J. Aggarwal R. Buyyarapu R. Kumpatla S. (2012). SNP markers and their impact on plant breeding. Int. J. Plant Genomics, 728398. doi: 10.1155/2012/728398 23316221 PMC3536327

[B37] Marçais G. Kingsford C. (2011). A fast, lock-free approach for efficient parallel counting of occurrences of k-mers. Bioinformatics 27, 764–770. doi: 10.1093/bioinformatics/btr011 21217122 PMC3051319

[B38] Morgil H. Gercek Y. C. Tulum I. (2020). “Single nucleotide polymorphisms (SNPs) in plant genetics and breeding,” In The recent topics in genetic polymorphisms Çalışkan M.. eds. (IntechOpen). doi: 10.5772/intechopen.91886

[B39] Naik K. C. (1949) South Indian fruits and their culture. South Indian fruits their culture P. Varadachary & Co., Madras. p. 335

[B40] Ohri D. Kumar A. (1986). Nuclear DNA amounts in some tropical hardwoods. Caryologia 39, 303–307. doi: 10.1080/00087114.1986.10797792

[B41] Ong B. Nazimah S. Osman A. Quek S. Voon Y. Hashim D. M.. (2006). Chemical and flavour changes in jackfruit (Artocarpus heterophyllus lam.) cultivar J3 during ripening. Postharvest. Biol. Technol. 40, 279–286. doi: 10.1016/j.postharvbio.2006.01.015

[B42] Ou S. Su W. Liao Y. Chougule K. Agda J. R. Hellinga A. J.. (2019). Benchmarking transposable element annotation methods for creation of a streamlined, comprehensive pipeline. Genome Biol. 20, 1–18. doi: 10.1186/s13059-019-1905-y 31843001 PMC6913007

[B43] Rahman A. M. Huq E. Mian A. Chesson A. (1995). Microscopic and chemical changes occurring during the ripening of two forms of jackfruit (Artocarpus heterophyllus l.). Food Chem. 52, 405–410. doi: 10.1016/0308-8146(95)93290-8

[B44] Rahman M. A. Nahar N. Mian A. J. Mosihuzzaman M. (1999). Variation of carbohydrate composition of two forms of fruit from jack tree (Artocarpus heterophyllus l.) with maturity and climatic conditions. Food Chem. 65, 91–97. doi: 10.1016/S0308-8146(98)00175-7

[B45] Rahman M. Patwary M. A. Barua H. Nahar S. Ahmmed A. N. F. (2016). Evaluation of yield and quality of three jackfruit (Artocarpus heterophyllus l.) genotypes. Agriculturists. 14, 107–111. doi: 10.3329/agric.v14i1.29108

[B46] Saha M. Saha M. Rahman M. Nazrul M. Quasem A. Halder N. (1996). “Variability in jackfruit,” in Proceedings of the Internal Research Review Workshop (Gazipur, Bangladesh: Horticulture Research Center, BARI), 1–4.

[B47] Sahu S. K. Liu M. Yssel A. Kariba R. Muthemba S. Jiang S.. (2020). Draft genomes of two artocarpus plants, jackfruit (*A. heterophyllus*) and breadfruit (*A. altilis*). Genes 11, 27. doi: 10.3390/genes11010027 PMC7017358 31878322

[B48] Seppey M. Manni M. Zdobnov E. M. (2019). BUSCO: assessing genome assembly and annotation completeness. Methods Mol. Biol. 1962, 227–245. doi: 10.1007/978-1-4939-9173-0_14 31020564

[B49] Siddiki A. Z. Baten A. Billah M. Alam M. Shawrob K. S. M. Saha S.. (2019). The genome of the black Bengal goat (Capra hircus). BMC Res. Notes 12, 1–3. doi: 10.1186/s13104-019-4400-3 31248431 PMC6598337

[B50] Sidhu A. S. (2012). Jackfruit improvement in the Asia-pacific region: A status report (APAARI) FAO Regional Office for Asia & the Pacific (FAO RAP) Maliwan Mansion, 39, Phra Atit Road Bangkok 10200.

[B51] Simmonds M. Preedy V. R. (2015). Nutritional composition of fruit cultivars (Academic Press). (San Diego, USA: Academic Press).

[B52] Somerville C. Koornneef M. (2002). A fortunate choice: the history of arabidopsis as a model plant. Nat. Rev. Genet. 3, 883–889. doi: 10.1038/nrg927 12415318

[B53] Statistics. Y. O. A. (2020). Bangladesh Bureau of statistics (BBS), statistics and informatics division (SID), ministry of planning, government of the people’s republic of Bangladesh (Government of the People’s Republic of Bangladesh).

[B54] Varshney R. K. (2010). “Gene-based marker systems in plants: high throughput approaches for marker discovery and genotyping,” in Molecular techniques in crop improvement (Dordrecht: Springer), 119–142. doi: 10.1007/978-90-481-2967-6_5

[B55] Vurture G. W. Sedlazeck F. J. Nattestad M. Underwood C. J. Fang H. Gurtowski J.. (2017). GenomeScope: fast reference-free genome profiling from short reads. Bioinformatics 33, 2202–2204. doi: 10.1093/bioinformatics/btx153 28369201 PMC5870704

[B56] Yamamoto Y. Y. Ichida H. Matsui M. Obokata J. Sakurai T. Satou M.. (2007). Identification of plant promoter constituents by analysis of local distribution of short sequences. BMC Genomics 8, 1–23. doi: 10.1186/1471-2164-8-67 17346352 PMC1832190

[B57] Zhang L. Lin G. Nino-Liu D. Foolad M. R. (2003). Mapping QTLs conferring early blight (Alternaria solani) resistance in a lycopersicon esculentum× l. hirsutum cross by selective genotyping. Mol. Breed. 12, 3–19. doi: 10.1023/A:1025434319940

[B58] Zhang Y. Li B. Xu F. He S. Zhang Y. Sun L.. (2021a). Jackfruit starch: Composition, structure, functional properties, modifications and applications. Trends Food Sci. Technol. 107, 268–283. doi: 10.1016/j.tifs.2020.10.041

[B59] Zhang W. Mirlohi S. Li X. He Y. (2018). Identification of functional single-nucleotide polymorphisms affecting leaf hair number in brassica rapa. Plant Physiol. 177, 490–503. doi: 10.1104/pp.18.00025 29686057 PMC6001316

[B60] Zhang Y. Wang Q. Zhang Y. Wu G. Tan L. Zhang Z. (2022). Effects of moisture content on digestible fragments and molecular structures of high amylose jackfruit starch prepared by improved extrusion cooking technology. Food Hydrocolloids. 133, 108023. doi: 10.1016/j.foodhyd.2022.108023

[B61] Zhang Y. Zuo H. Xu F. Zhu K. Tan L. Dong W.. (2021b). The digestion mechanism of jackfruit seed starch using improved extrusion cooking technology. Food Hydrocolloids. 110, 106154. doi: 10.1016/j.foodhyd.2020.106154

