# Supplementary material for: Whole-genome sequencing of a year-round fruiting jackfruit (Artocarpus heterophyllus Lam.) reveals high levels of single nucleotide variation
Source: Front Plant Sci. 2022 Dec 20;13:1044420. doi: 10.3389/fpls.2022.1044420 (PMC9809283; doi:10.3389/fpls.2022.1044420)
Supplement: Supplementary Table 2 — Flowering locus C (FLC). Listing of flowering gene orthologues (Enclosed in a separate file). [file Table_2.docx]

**Table S2:** Comparison between the previously reported *Artocarpus heterophyllus* sequence and BARI Kanthal-3 sequence

| **Parameters** | **Previously reported *A. heterophyllus* sequence** | **BARI Kanthal-3 sequence** |
| --- | --- | --- |
| 1. No. of scaffolds | - 1. M scaffolds totaling 982 Mb | 218,562 scaffolds totaling 843 Mb |
| 1. Size of N50s in the scaffolds | 548 kb with the longest being 3.1 Mb | 425 kb and with the longest being 2.6 Mb |
| 1. GC content (%) | 32.9 % | 34.10%. |
| 1. BUSCO results | 1369 (95%) complete BUSCOs where 932 (64.7%) BUSCO genes were “complete single-copy”, 437 (30.3%) were “complete duplicated”, 15 (1%) were “fragmented”, and 56 (4%) were “missing” | 1569 (97.2%) were complete BUSCOs in which 1094 (67. 8%) BUSCO genes were “complete single-copy”, 475 (29.4%) were “complete duplicated”, 21 (1.3%) were “fragmented”, and 24 (1.5%) were “missing” |
| 1. Genome size | The estimated genome size was 1.01 Gbp | The estimated genome size was 1.04 Gbp |
