# Supplementary figures and images for: Whole-genome sequencing of a year-round fruiting jackfruit (Artocarpus heterophyllus Lam.) reveals high levels of single nucleotide variation
Source: Front Plant Sci. 2022 Dec 20;13:1044420. doi: 10.3389/fpls.2022.1044420 (PMC9809283; doi:10.3389/fpls.2022.1044420)

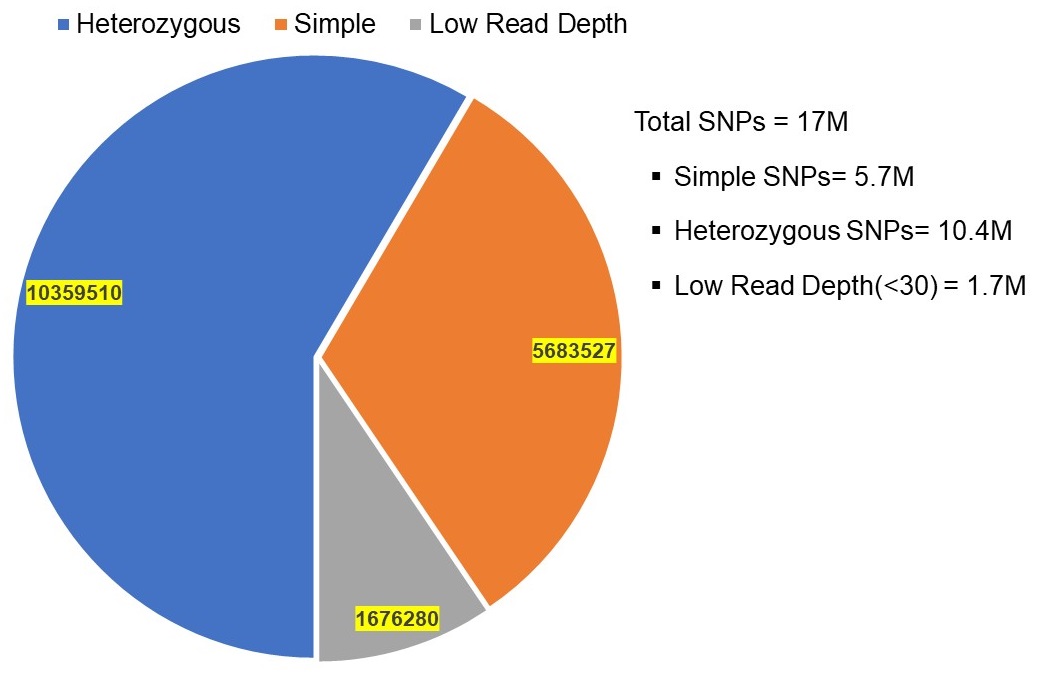

Supplement: Supplementary Figure 1 — Categories of candidate SNPs identified in the genome of BARI_Kanthal-3. [file Image_1.jpg]
